# Parental perspectives on physical inactivity and unhealthy diet as linked Non-Communicable Disease (NCD) risk factors among adolescents in Coastal Karnataka, India: a qualitative study

**DOI:** 10.1186/s12889-026-28158-7

**Published:** 2026-06-22

**Authors:** Revati Amin, Muralidhar M. Kulkarni, Shivashankara Kaniyoor Nagri, Ravindra Neelakanthappa Munoli, Vennila J., Suvarna Hebbar, Prateek Srivastav, Tulasiram Bommasamudram, R. Vishnu Vardhan, Shishira K. B., Naga Padmaja K. S., Ashwini Pai K., Rutuja S. Rao, Pooja Shetty, Chaitra, Lavanya Nayak, K. Vaishali

**Affiliations:** 1 Department of Physiotherapy, Kasturba Medical College Mangalore, Manipal Academy of Higher Education, Manipal, India; 2 Department of Community Medicine, Kasturba Medical College, Manipal Academy of Higher Education, Manipal, Karnataka India; 3 Department of Medicine, Kasturba Medical College, Manipal Academy of Higher Education, Manipal, Karnataka India; 4 Department of Psychiatry, Kasturba Medical College, Manipal Academy of Higher Education, Manipal, Karnataka India; 5 Manipal College of Health Professions, Manipal Academy of Higher Education, Manipal, India; 6 Department of Clinical Nutrition and Dietetics, Manipal College of Health Professions, Manipal Academy of Higher Education, Manipal, Karnataka India; 7 Department of Physiotherapy, Manipal College of Health Professions, Manipal Academy of Higher Education, Manipal, Karnataka India; 8 Department of Exercise and Sports Sciences, Manipal College of Health Professions, Manipal Academy of Higher Education, Manipal, Karnataka India; 9 Department of Statistics, Pondicherry University (A Central), Puducherry, 605014 India

**Keywords:** Adolescents, Physical inactivity, Diet, Parents, Non-communicable diseases, Qualitative research

## Abstract

**Background:**

Unhealthy dietary practices and physical inactivity during adolescence are key contributors to the future burden of non-communicable diseases (NCDs). Although parental influence on adolescents’ lifestyle behaviours is well recognized, evidence from low- and middle-income countries (LMICs), particularly within context-specific settings, remains limited. This study aimed to explore parents’ perspectives on physical inactivity and unhealthy dietary practices as inter-related risk factors for NCDs among adolescents in Coastal Karnataka, India.

**Methods:**

This qualitative study employed semi-structured interviews with 16 parents (10 mothers and 6 fathers) of adolescents aged 11–17 years in Coastal Karnataka, India. Participants were purposively recruited to ensure variation across school types and socio-economic backgrounds. Interviews were audio-recorded, transcribed verbatim, and analysed using a hybrid inductive–deductive thematic approach informed by the Capability–Opportunity–Motivation Behaviour (COM-B) model, Social Cognitive Theory, and the Theory of Planned Behaviour.

**Results:**

Six interrelated themes were identified: (1) sustained awareness of healthy behaviours with a persistent awareness–practice gap; (2) the home environment as a space of negotiation rather than control; (3) normalization of intermittent unhealthy dietary practices and episodic physical inactivity; (4) academic demands as key barriers to healthy lifestyles; (5) schools as conditional but time-limited protective environments; and (6) gendered and culturally embedded influences on physical activity. Despite high levels of awareness, parents reported increasing difficulty in sustaining healthy behaviours due to academic pressures, adolescents’ autonomy, and environmental constraints.

**Conclusions:**

Parental recognition of the importance of healthy behaviours is constrained by structural, academic, and socio-cultural factors that limit sustained adoption. Interventions targeting adolescent NCD prevention in LMIC settings should adopt multi-level strategies that integrate family support, school-based initiatives, and context-specific considerations such as academic pressures and cultural practices.

**Trial registration:**

Clinical Trials Registry of India (CTRI/2025/05/087338), dated: 14/05/2025.

**Supplementary Information:**

The online version contains supplementary material available at https://doi.org/10.1186/s12889-026-28158-7.

## Background

Non-communicable diseases (NCDs) are the leading cause of premature death globally with over 70% of global deaths attributable to NCDs; however, an ever-increasing proportion of those deaths occur in Low- and Middle-Income Countries (LMICs) [1, 2]. As two of the most important modifiable behavioral risk factors that contribute to the burden of NCDs, physical inactivity and unhealthy dietary habits are associated with a substantial portion of cases of obesity, diabetes, cardiovascular disease, and other metabolic disorders [1, 3]. Importantly, many of these behaviors begin in adolescence and remain relatively stable throughout adulthood, thus establishing long-term health trajectories for individuals across their lifespan [4, 5].

More than 80% of adolescents (ages 11–17) are not meeting recommended levels of physical activity (PA) and there is a growing trend towards diets characterized by increased consumption of energy-dense and ultra-processed foods [4, 6]. There is evidence suggesting that both physical inactivity and unhealthy dietary habits commonly co-exist and interact to increase cumulative risk for NCDs rather than being independent risk behaviors [7, 8]. Because adolescence is a formative developmental stage in which established lifestyle behaviors can either mitigate or worsen the long-term burden of non-communicable diseases (NCD), the adolescent years represent a key opportunity for the prevention of NCD. With regard to NCD burden in India, there has been a rapid epidemiological transition to a predominantly NCD-driven pattern of mortality, with NCDs accounting for more than 60% of all deaths nationally [2]. Adolescents in India are increasingly exposed to sedentary lifestyles due to academic pressure, extended periods of screen time, and changes in their food environment, all of which undermine opportunities for regular PA and healthy eating [9–11]. Consistent with previous research conducted in various parts of India, national and regional studies have reported similarly low levels of PA and poor-quality dietary habits among Indian adolescents, particularly in urbanizing and academically competitive areas [9, 12]. Notwithstanding this fact, the majority of NCD prevention initiatives in India have been directed toward adults with limited attention to the upstream behavioral determinants of NCDs in adolescence.

In the larger national context described above, Coastal Karnataka provides a unique socio-cultural and environmental setting that merits specific examination. This region has experienced rapid increases in education attainment and high parental expectations for academic achievement, mixed urban-semi rural environments and a rich cultural heritage that includes traditional dietary practices and indigenous forms of PA (e.g., yoga, dance, and martial arts) [13, 14]. However, the increasing exposure to modern sedentary lifestyles, private tuition culture, and dietary changes place adolescents in this region at higher risk for physical inactivity and unhealthy eating. Empirical evidence assessing how families in this region understand and manage these risks is limited.

Parents exert considerable influence on the lifestyle behaviors of their adolescents through the provision of food availability in the home, establishment of household norms, role modeling, encouragement, and regulation [15, 16]. However, adolescence is also characterized by increasing autonomy, academic demands, and social influences that reduce the ability of parents to maintain control and promote sustained health-enhancing behaviors [17]. Quantitative studies have documented associations between parent’s practices and the behavior of adolescents; however, these studies offer very limited information regarding how parents perceive the challenges and competing demands that affect their capacity to engage in health-enhancing behaviors, and how they adjust their roles in daily contexts [15, 16].

Studies using qualitative designs in high income countries indicate that parents often face conflict between promoting healthy behaviors and accommodating academic pressures, social expectations, and adolescent preferences when making decisions about what types of diets and amounts of PA are acceptable [8, 18]. Similar qualitative studies from low-middle income countries and specifically from diverse cultures in India, such as Coastal Karnataka, are limited. Recent qualitative studies conducted in the Indian context have explored adolescents’ dietary behaviours and the role of family and school environments in shaping these behaviours. For instance, a qualitative study from urban India reported that adolescents’ food choices are strongly influenced by taste preferences, peer interactions, and the increasing availability of processed foods, despite awareness of healthy eating practices [12]. Similarly, studies examining the perspectives of parents and teachers in India have highlighted concerns regarding the adequacy of adolescents’ diets and the challenges faced in promoting healthy eating within the constraints of academic pressures and changing food environments [19]. Evidence from broader LMIC settings further indicates that parental decision-making regarding adolescents’ diet and physical activity is influenced by a complex interplay of cultural norms, socioeconomic factors, and environmental constraints [8, 18].

However, these studies are largely context-specific and do not adequately capture the unique socio-cultural, academic, and environmental dynamics present in Coastal Karnataka. In particular, the interplay between high academic expectations, evolving dietary patterns, and culturally embedded physical activity practices in this region remains underexplored. Therefore, there is a need for context-specific qualitative research to better understand how parents in Coastal Karnataka perceive and manage adolescents’ lifestyle behaviours linked to NCD risk.

This limitation is important because of the ways in which sociocultural norms, gender expectations, safety concerns, and academic pressures create a unique set of circumstances that shape the decision-making process of parents in terms of whether to intervene and how feasible it will be to support their adolescent’s adoption of healthy behaviors [4, 11, 13].

Additionally, there is a need for greater knowledge about how academic pressures and cultural norms jointly influence parents’ decision-making processes in semi-urban and urbanizing regions of India. To guide the conceptual orientation of this study, an integrated theoretical framework drawing on the Capability–Opportunity–Motivation Behaviour (COM-B) model, Social Cognitive Theory (SCT), and the Theory of Planned Behaviour (TPB) was adopted. The COM-B model provides a comprehensive structure to understand behaviour through the interaction of capability, opportunity, and motivation [20]. SCT emphasizes the role of social and environmental influences, including observational learning and family dynamics, in shaping behaviours [21]. TPB further contributes by explaining how attitudes, subjective norms, and perceived behavioural control influence behavioural intentions [22].

The integration of these frameworks allows for a multi-level understanding of adolescents’ lifestyle behaviours by capturing individual, interpersonal, and environmental determinants. This theoretical grounding informed the development of the interview guide and supported a more comprehensive exploration of parents’ perceptions regarding physical inactivity and unhealthy dietary practices among adolescents.

As a result, this study was designed to investigate parents’ views on physical inactivity and unhealthy dietary practices as inter-related risk factors for NCDs among adolescents in Coastal Karnataka, India. The aim of the study was to investigate parents’ views on physical inactivity and unhealthy dietary practices as risk factors for NCDs among adolescents. The objectives of the study were (1) to evaluate parents’ understanding of PA and diet in relation to the health of adolescents and prevention of NCDs. (2) to investigate perceived facilitators and barriers to maintaining healthy lifestyle behaviors during adolescence. (3) to determine the influences of sociocultural, academic, and environmental factors on parental decision-making.

## Methods

This qualitative study was conducted between May and August of 2025 in three school blocks (Udupi, Karkala, and Kundapura) of Udupi District, Coastal Karnataka, India. The study formed Phase 1 (formative qualitative component) of a larger cross-sectional study aimed at understanding lifestyle-related risk factors among adolescents. Ethical approval was obtained from the Institutional Ethics Committee of Kasturba Medical College and Kasturba Hospital, Manipal Academy of Higher Education (IEC No. 413–2024). The study was also registered with the Clinical Trials Registry of India (CTRI/2025/05/087338) dated: 14/05/2025. Written informed consent was obtained from all participants prior to data collection. The study adhered to the Declaration of Helsinki.

The study was reported in accordance with the Consolidated Criteria for Reporting Qualitative Research (COREQ) checklist [23], and the completed checklist with page references is provided as Supplementary File 1.

Participants consisted of parents or primary caregivers of adolescents aged 11–17 years (grades 5–10) attending public or private schools in the three selected school blocks. Parents of adolescents without serious medical conditions and who were willing to participate in in-depth semi-structured interviews were eligible for inclusion. Purposive sampling was employed to ensure diversity with respect to adolescents’ age, gender, school type, and parental socioeconomic status. Sampling was guided by the principle of thematic saturation, whereby recruitment continued until no new concepts emerged from successive interviews, consistent with established qualitative research methods [24]. This approach is widely used to ensure adequate depth and comprehensiveness of qualitative data. A total of 16 parents participated in the study.

Multiple recruitment strategies were employed to enhance reach while maintaining geographic specificity. Digital dissemination of study flyers was done through social media platforms (WhatsApp, Facebook, and Instagram), targeting parents residing in the three school blocks through local community groups, school-affiliated networks, and researchers’ professional contacts within Udupi District. This approach was used to complement, not replace, location-based recruitment. Printed flyers were displayed on notice boards of government and private schools across the three school blocks, with support from teaching staff to identify and invite eligible parents. Flyers were also placed in commonly frequented community locations. Additionally, snowball sampling was used, whereby enrolled participants referred other eligible parents from their personal networks.

Data was collected through semi-structured interviews conducted either in person or telephonically, based on participant preference and logistical feasibility. All interviews were conducted in English, Kannada, or bilingually. Prior to the interviews, participants were informed about the study objectives, procedures, confidentiality safeguards, and their right to withdraw at any time. Written informed consent was obtained for in-person interviews, and audio-recorded verbal consent was obtained for telephonic interviews, in accordance with ethics committee approval.

Interviews lasted approximately 45–60 min and were conducted by a licensed and certified physiotherapist trained in qualitative interviewing. Interviews were audio-recorded with participant permission and anonymized to protect confidentiality.

An interview guide was developed based on the Capability–Opportunity–Motivation Behaviour (COM-B) model, Social Cognitive Theory (SCT), and the Theory of Planned Behaviour (TPB) [20–22]. The integration of these frameworks was undertaken to ensure a comprehensive and multi-level exploration of factors influencing adolescents’ lifestyle behaviours. The COM-B model served as the primary organizing framework to identify behavioural determinants by examining capability (knowledge and skills), opportunity (physical and social environment), and motivation (beliefs, intentions, and habits) required for behaviour change [20]. SCT complemented this approach by capturing the influence of social interactions, observational learning, and environmental context, particularly within family and school settings [21]. The TPB further informed the exploration of behavioural intentions by focusing on attitudes, subjective norms, and perceived behavioural control [22].

To address conceptual overlap between these frameworks, constructs were operationalized in a complementary manner rather than treated as independent domains. Specifically, COM-B provided the overarching structure for organizing interview domains, while SCT and TPB informed the development of probing questions related to social influence, behavioural intentions, and perceived control. This integrated approach ensured that individual, interpersonal, and environmental determinants of behaviour were explored systematically while maintaining conceptual clarity.

The combined use of these theoretical frameworks was intended to provide a comprehensive understanding of the multi-level factors influencing adolescents’ lifestyle behaviours. The COM-B model was used to identify key behavioural determinants by examining individuals’ capability (knowledge and skills), opportunity (environmental and social context), and motivation (beliefs and intentions) required for behaviour change [20]. SCT complemented this by addressing the role of social influences, observational learning, and environmental factors in shaping behaviours, particularly within family and school contexts [21]. The TPB further informed the exploration of behavioural intentions by focusing on attitudes, subjective norms, and perceived behavioural control [22].

These frameworks were integrated to ensure that the interview guide captured individual, interpersonal, and environmental dimensions of behaviour. While conceptual overlap exists between the models, each framework contributed distinct but complementary perspectives, thereby enhancing the depth and comprehensiveness of data collection. The interview questions were therefore structured to explore parents’ perceptions across these domains in a coherent and theoretically informed manner.

The guide explored parents’ understanding and perceptions of their adolescents about (a) PA (including yoga) and dietary patterns; (b) parents’ roles, barriers and facilitators towards their adolescents in engaging them in PA and/or making healthy food choices; (c) how schools influence their adolescents’ PA levels and diet; and (d) socio-cultural factors.

Content validity of the interview guide (supplementary file) was assessed by a panel of six experts (two physicians, two yoga therapists, and two physiotherapists), each with a minimum of five years of professional experience. The guide was subsequently pilot tested with ten parents (not included in the final sample), and minor refinements were made based on feedback. Experts agreed that the interview questions were conceptually aligned with COM-B, SCT, and TPB frameworks [20–22]. The guide was used to sensitize inquiry, and probing questions were employed only when clarification or elaboration was required.

### Transcription, translation, and data analysis

All interviews were transcribed verbatim. Kannada transcripts were translated into English by bilingual researchers fluent in both languages, and translations were cross-checked for accuracy. Demographic data were summarized using descriptive statistics in Jamovi (version 2.2.5).

Thematic analysis was conducted using a hybrid inductive–deductive approach. Two researchers (VK and RA) independently performed line-by-line coding of the transcripts. Deductive coding was guided by COM-B, SCT, and TPB [20–22] to provide an analytic framework, while inductive coding allowed for the emergence of novel themes. Coding discrepancies were resolved through discussion, and consensus was achieved through iterative team review.

A constructivist orientation within a critical realist framework informed analysis. Codes were organized into categories through axial coding, followed by selective coding to identify relationships and develop overarching themes. A detailed codebook was maintained, linking codes to transcript excerpts. Two-phase thematic mapping was undertaken: first at the level of individual interviews, and subsequently across the entire dataset to refine themes and deepen interpretive analysis. Analytical procedures followed the guidance of Braun and Clarke [25], emphasizing interpretation beyond surface description.

### Research team and reflexivity

The investigators who designed this project are from various disciplines as listed above. The conceptualization for this project was developed by the investigators all of whom have experience in adolescent health, public health and qualitative research.

The investigator, RA, is a female Physiotherapist with a PhD that has prior experience in qualitative research and had received formal training in conducting semi structured interviews. RA was able to conduct both interviews in English and Kannada, allowing her to communicate effectively, understand the context and obtain accurate data.

Coding and theme development were completed separately by RA and KV. However, each researcher reviewed emerging themes, coding decision making and interpretation with the rest of the multi-disciplinary research team. This included several specialists such as community medicine, psychiatry, nutrition and physiotherapy. Therefore, while developing these analyses there was an opportunity to critically reflect on the analyses and to avoid discipline specific biases.

During this project, the investigators kept record of reflexive discussions and memo’s to identify possible influences from their background as practitioners or previous assumptions when interpreting the results. All disagreements in interpretation were resolved by discussing them until agreement was reached; therefore, all three criteria for establishing credibility, trustworthy and reflexivity according to COREQ were met.

No prior personal relationship was established between the interviewer and participants before recruitment. Participants were informed about the purpose of the study, the role of the researcher, and the voluntary nature of participation prior to obtaining consent. This helped to minimize potential power dynamics and encourage open and honest responses.

Reflexivity was maintained throughout the research process to acknowledge and minimize potential researcher bias. The interviewer maintained reflexive field notes after each interview to document observations, contextual factors, and personal reflections. Regular debriefing sessions were conducted with the research team to discuss emerging findings, address subjective interpretations, and ensure analytical rigor. The multidisciplinary research team, comprising experts in physiotherapy, medicine, nutrition, and public health, contributed to diverse perspectives during data interpretation, thereby enhancing credibility and confirmability of the findings.

### Rigor and trustworthiness

Member checking was not performed because interviews focused on collective perceptions rather than individual experiences; however, investigator triangulation and peer debriefing were used to enhance interpretive validity. Dependability was ensured by maintaining an audit trail, including rationality, documenting analytic decisions. Transferability was supported through rich description of the study context and participant characteristics.

## Results

A total of 16 parents participated in the study, including 10 mothers and 6 fathers. The mean age of participants was 48 ± 16 years. Participants had varied educational backgrounds, with the majority having completed secondary or higher education. In terms of school type, 9 participants had adolescents attending government (public) schools and 8 participants had adolescents attending private schools. Interviews were conducted via telephone (*n* = 16), based on participant preference and feasibility. A summary of participants’ socio-demographic characteristics is presented in Table 1.

**Table 1:** Demographic details

Variable	Category	*n* (%)
Sex	Mothers	10 (62.5)
	Fathers	6 (37.5)
Age (years)	Mean ± SD	48 ± 16
School type	Government (Public)	9
	Private	8
Mode of interview	Face-to-face	0
	Telephone	16

Following the description of participant characteristics, analysis of interviews with 16 parents revealed six major themes that collectively illustrated how parents’ perceptions of adolescents’ physical inactivity and consumption of unhealthy diets are developing as interconnected risk factors for future NCD during adolescence. (Table 2) To maintain readability and avoid redundancy, illustrative quotes are primarily presented in the main text, with additional supporting quotes provided in Supplementary File 2.

**Table 2:** Themes, subthemes, and illustrative quotes reflecting parents’ perspectives on physical inactivity and unhealthy diet as NCD risk factors among adolescents

Theme	Subtheme
1. Awareness of healthy lifestyle and NCD prevention (Sustained awareness of healthy behaviours but with prevailing awareness-practice gap)	1.1 Recognition of PA and diet as essential for health
	1.2 Awareness–practice gap
2. Home environment as primary space of regulation and negotiation	2.1 Preference for home-cooked, traditional food
	2.2 Negotiation rather than strict control
3. Normalisation of ‘occasional’ unhealthy diet and episodic inactivity (Normalization of intermittent unhealthy eating practices and intermittent physical inactivity)	3.1 Junk food framed as rare and acceptable
	3.2 Acceptance of inactivity during busy periods
4. Academic pressure reshaping daily routines (Academic demands as barriers to healthy lifestyles)	4.1 Decline in PA with increasing age
	4.2 Academic schedules disrupting eating patterns
5. School environment as a conditional protective factor	5.1 Trust in school meals and physical education
	5.2 Reduced effectiveness in higher grades
6. Gendered and cultural influences on lifestyle behaviours	6.1 Gender-related constraints on PA
	6.2 Cultural practices recognised but under-utilised

### Theme 1: high awareness of healthy lifestyles and NCD prevention (sustained awareness of healthy behaviours but with prevailing awareness-practice gap)

#### Subtheme 1.1: recognition of healthy dietary practices and physical activity as foundational to health

Parents recognized diet and PA as fundamental determinants of health and key to the prevention of future lifestyle diseases, including obesity and diabetes. Parents believed that PA promotes fitness, reduces stress, and regulates emotions, whereas diet was linked to strength, immunity, and sustained long-term health.

*“Exercise keeps them active physically…mentally it is good too.” M7*.

*“Eating outside food will ruin their health.” F2*.

#### Subtheme 1.2: awareness is insufficient to promote consistent and sustainable behavior

Despite high levels of awareness regarding the importance of healthy lifestyles, parents had reported of difficulty in consistently encouraging and sustaining healthy lifestyle and behaviours in their adolescents. Parents identified that compromises in their adolescents’ diets and PA levels were often due to competing interests, their adolescents’ preferences, and situational constraints.

*“We do eat junk food in the evenings…we do not always eat healthy.” M1*.

These accounts illustrate a disconnect between parental knowledge and the establishment of supportive routines. Although parents had a high level of awareness of the importance of healthy diet/eating and regular PA for adolescents, they reported limited success in fostering consistent habits that would sustain adolescents’ engagement in these.

### Theme 2: the home environment as a space of negotiation rather than control

#### Subtheme 2.1: home-cooked meals as a protective measure

Parents viewed the home environment as a protective space against unhealthy dietary practices. Parents identified that preparing meals at home utilizing traditional ingredients and recipes such as rice, dhal, vegetables, idly, dosa, chapatti, and curries were beneficial to their adolescents’ health and protective against future NCDs.

*“Most of what he eats is prepared at home.“M7*.

Furthermore, parents expressed a sense of responsibility for preparing healthy meals for their adolescents, especially during early adolescence, when adolescents begin to assert more independence from their families. Home-cooked food was thus framed as both a nutritional safeguard and a parental duty.

#### Subtheme 2.2: regulating food and encouraging PA through negotiation rather than enforcement

As adolescents transitioned beyond childhood, parents relied on variety of negotiation methods rather than enforcement to regulate dietary intake and encourage PA. Parents stated that negotiating with their adolescents allowed them to tolerate occasional unhealthy foods and decreased activity levels. While negotiation was perceived as developmentally appropriate, the normalization of episodic unhealthy dietary behaviors and inactivity may contribute cumulatively to increased chronic disease risk over time.

*“During the holidays*,* I do not get to supervise him as much.“M6*.

Similarly, parents noted that encouraging PA through direct supervision became less frequent as their adolescents gained more autonomy, had increased access to school canteens, and had less time allocated to physical education programs.

*“Therefore*,* I give her more freedom and allow her to decide which activities she wants to participate in.“F9*.

### Theme 3: normalisation of occasional unhealthy diet and episodic inactivity (normalization of intermittent unhealthy eating practices and intermittent physical inactivity)

#### Subtheme 3.1: unhealthy food framed as rare, social, and acceptable

Parents frequently minimize the perceived health risks of unhealthy foods such as junk foods, bakery products, sweets, and fast foods as being consumed *“occasionally*,*” “once per month*,*”* or *“only at festivals”.*

*“Junk food is consumed by my son maybe every other month*,* and that’s the extent of his consumption.“F3*.

Parents similarly normalized episodic physical inactivity as a natural consequence of academic demands and social obligations, further reducing parental concern regarding its long-term health implications.

### Theme 4: academic demands as barriers to healthy lifestyles

#### Subtheme 4.1: decrease in PA with increasing age and academic load

Parents consistently stated that younger adolescents were naturally more active and had fewer academic commitments, and thus were able to engage in regular PA. However, as adolescents advanced in age, PA declined due to increasing academic demands of their matriculation.

*“My daughter has been extremely busy with her studies*,* so she does not have much time to engage in physical activity.”*

*“The younger one is more active…she enjoys playing outside.” M5*.

*“The older one is too busy with schoolwork and attending college classes.“M3*.

#### Subtheme 4.2: academic schedule disruptions to regular mealtimes

In addition, parents stated that the extended school day, additional tutoring classes, and homework disrupted the normal routine of mealtimes, resulting in irregularities in meal timing, increased snacking, and reduced parental ability to monitor their adolescents’ dietary intake. Therefore, parents suggested that academic stressors were a common factor influencing both dietary habits and PA level among adolescents.

*“It’s hard to find the time to prepare healthy meals after a long day at school.“M1*.

### Theme 5: schools as conditional but time-limited protective environments

#### Subtheme 5.1: trust in school meals and physical education

Parents expressed considerable trust on schools as institutions that would protect their adolescents from engaging in unhealthy behaviors and provide their adolescents with opportunities to engage in healthy behaviors such as eating healthy school meals and participating in physical education programs.

*“I trust that the school food is healthy… rice*,* sambar*,* vegetables*, etc.,*“M5*.

*“In school*,* they have yoga*,* karate*,* and sports programs… she participates in all of them.“F7*.

#### Subtheme 5.2: diminishing effectiveness of school protection in higher grades

Despite this trust, parents acknowledged that schools could not continue to protect their adolescents’ health indefinitely. As adolescents grow older, they become more independent, have more access to canteen foods, and have less time devoted to physical education.

*“This trend is evident in the upper secondary school grades.“F9*.

Therefore, parents suggested that adolescence represents a developmental period during which external controls over behaviour become progressively less effective.

### Theme 6: gendered and cultural influences on lifestyle behaviours

#### Subtheme 6.1: gender-specific constraints on PA

Parents acknowledged that there were limitations to the degree to which boys and girls could engage in PA, particularly in terms of vigorous or outdoor activities.

*“For girls*,* wearing heavy Yaksagana costumes makes it difficult for them to perform.“F3*.

Parents indicated that household responsibilities, safety concerns, and social expectations placed constraints on girls’ participation in vigorous and outdoor activities.

#### Subtheme 6.2: cultural practices acknowledged but under-utilised

Parents recognized the value of traditional diets and cultural physical activities (e.g., yoga, dance forms, martial arts, Yaksagana) as physically demanding and health-promoting.

*“Yaksagana requires a great deal of physical strength and stamina.“M5*.

**Fig. 1:**
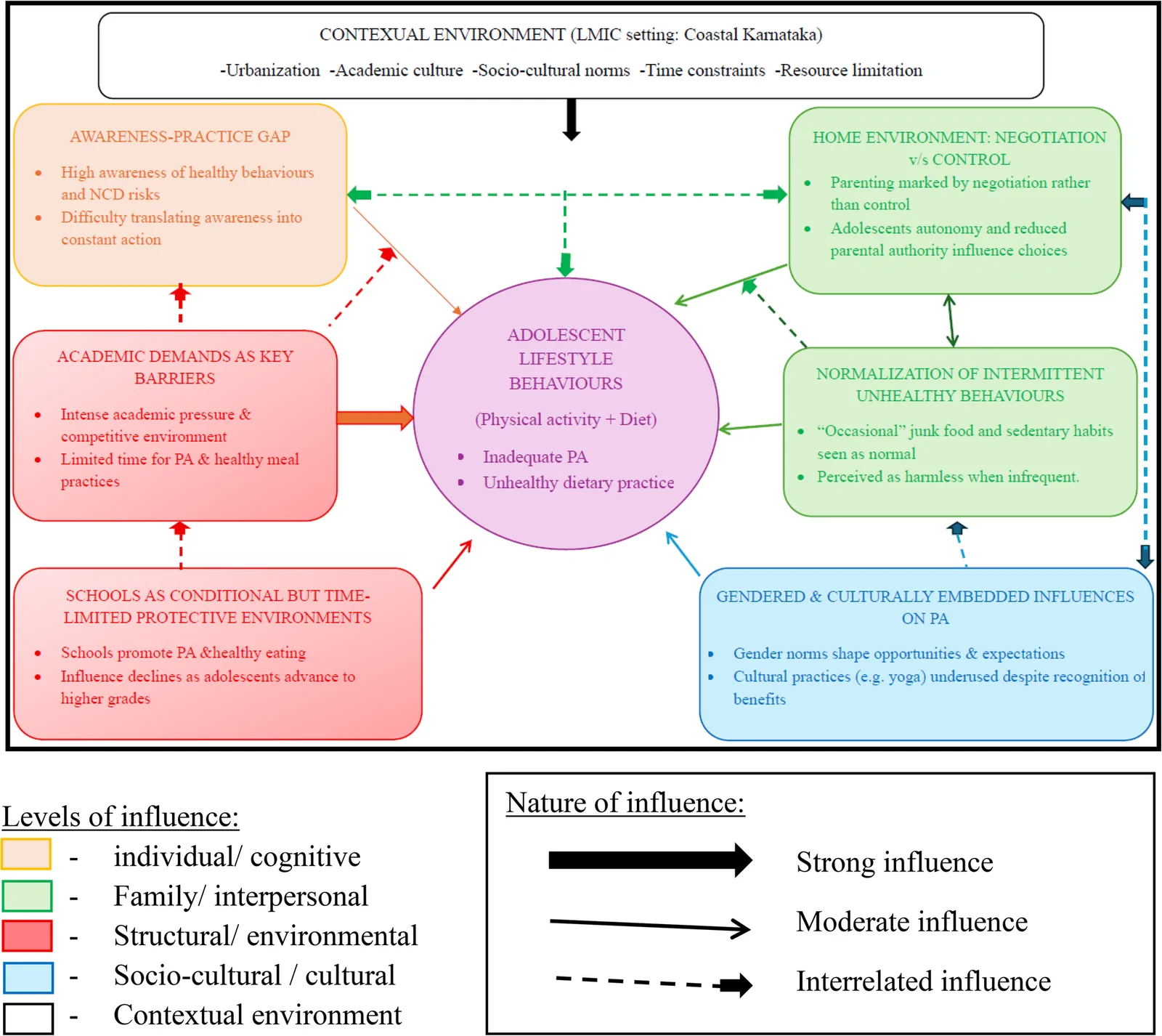
The conceptual map shows the interaction between family, individual, structural and socio-cultural factors within the broader contextual environment to shape adolescent lifestyle behaviours

Parents further acknowledged that adolescents had limited time and interest in participating in these culturally based activities. Therefore, parents identified a lost opportunity to utilize culturally grounded approaches to prevent NCD during adolescence. (Fig. 1)

## Discussion

This qualitative study sought to explore parental perceptions of physical inactivity and poor dietary behaviors as inter-related, yet distinct risk factors for NCDs among adolescents within a developing LMIC context (India). The study findings indicate a persistent awareness-practice gap among parents, not due to a lack of knowledge, but due to social structural, educational, and cultural barriers that are exacerbated when adolescents progress to higher academic grades, during which increasing autonomy, academic pressures, and reduced external regulation collectively undermine parents’ capacity to sustain healthy lifestyle behaviours.

### Awareness–practice gap in adolescent lifestyle behaviors

Parents were aware of the importance of physical activity and healthy dietary practices for preventing future chronic diseases. Similar findings have been reported in qualitative studies from India and other LMIC settings, where awareness of healthy behaviours was generally high, but implementation was constrained by environmental, social, and academic factors [12, 19]. However, this awareness did not consistently translate into healthy behaviours among either parents or adolescents.; this gap is supported by research indicating that simply being knowledgeable does not promote behavioral change, especially in adolescence, a developmental period characterized by competing priorities, increasing autonomy, and multiple environmental constraints [7]. This finding is consistent with evidence from the Indian context, where studies have reported that although adolescents and parents demonstrate awareness regarding healthy dietary practices and the importance of physical activity, this knowledge often does not translate into sustained healthy behaviours [12, 19]. Qualitative studies conducted in urban Indian settings have highlighted that factors such as taste preferences, peer influence, easy availability of processed foods, and competing academic priorities contribute to this disconnect between knowledge and practice. Additionally, parental perspectives in India indicate that while there is recognition of the importance of healthy lifestyles, practical challenges such as time constraints, academic pressures, and adolescents’ autonomy limit the consistent implementation of these behaviours [19].

These findings reinforce that the awareness–practice gap observed in the present study is not unique but reflects a broader pattern within the Indian socio-cultural context. However, our findings extend existing evidence by highlighting how this gap is further intensified in Coastal Karnataka due to the combined influence of high academic expectations, evolving dietary environments, and sociocultural norms that shape parental decision-making.

From a behavioral science viewpoint, this indicates that there were limitations in terms of opportunity and perceived behavioral control, as indicated in the Capability–Opportunity–Motivation Behaviour (COM-B) model and the Theory of Planned Behavior [20, 22]. Although parents had positive attitudes and motivation toward supporting healthy behavior, parents felt they did not have enough time, due to their adolescents’ increasing academic demands, and their adolescents’ preferences impacted parents’ ability to consistently support their adolescents in practicing healthy behaviors.

### Home environment and transition from control to negotiation

The home environment represented a significant space for both protection and compromise in shaping adolescents’ dietary and PA behaviours. Parents consistently valued home-cooked, traditional diets, which was consistent with previous evidence that traditional diets can provide protection against chronic disease risks [6, 26]. However, as adolescents aged, parents relied on negotiation rather than enforcing rules and regulations concerning food choices and activity routines. Similar findings have been identified in longitudinal and qualitative studies demonstrating that parental authority and control over adolescents’ lifestyle behaviors decreases as adolescents develop greater autonomy [15, 17] while negotiation allows adolescents to develop independence, repeated compromises can potentially normalize unhealthy behaviors.

### Normalization of “occasional” unhealthy behaviors

Parents regularly referred to unhealthy diet and inactivity as infrequent and acceptable to society. Other studies, from both high-income and low-middle-income countries have identified that parents tend to downplay the long-term effects of infrequently engaging in unhealthy behaviors [8, 18]. However, studies indicate that even sporadic engagement in unhealthy behaviors can lead to increased likelihood of developing sedentary lifestyles, excessive weight gain, and metabolic risk in adolescents [5, 27].

### Academic pressure as an upstream determinant

Academic pressure emerged as the most prominent upstream factor that affected the daily routines of adolescents. Parents consistently ranked academic performance above PA, especially in the later years of their adolescent’s academic career. Previous studies have identified that academic competition has been the primary obstacle to adolescent PA in India and South Asia [10, 11] reducing opportunities for PA in these developmental stages may negatively affect future health trajectories. In addition to limiting opportunities for PA, academic pressures also led to irregular meal times, and thus indirectly, limited the quality of the food adolescents ate. The results of this study demonstrate the need to address academic structures as part of comprehensive adolescent chronic disease prevention strategies.

### Schools as protective but inadequate environments

Parents perceived that schools provided an important structure for facilitating healthy behaviors through physical education, sports, yoga, and providing regular meals. Studies have demonstrated that school-based programs can increase PA in adolescents, particularly in areas where parents do not monitor their adolescents [28]. However, the decreased availability of physical education programs in higher grade levels parallels declines seen globally in physical education programs in secondary schools [29]. Therefore, protecting and incorporating physical education into the curriculum of senior academic levels may be crucial to achieving long-term success.

### Gendered and cultural factors affecting PA

Although parents expressed support for equal rights for boys and girls, subtle constraints existed for girls related to PA. Previous studies have documented safety concerns, household responsibilities, and social norms as barriers to girls’ PA in India and other low-middle-income countries [4, 13]. Recognizing the cultural significance of practices like yoga and traditional dance as healthy behaviors offers opportunities for culturally sensitive interventions. Incorporating culturally relevant practices like yoga and traditional dance into school and community-based programs may make them more acceptable and sustainable [14].

### Implications for policy and practice

The findings emphasize the importance of using culturally specific, adolescent-focused, and educationally relevant chronic disease prevention strategies for adolescents in low-middle-income countries. Interventions must consider regional sociocultural and educational realities in addition to addressing the structural feasibility of the interventions. Examples of parent-supported and school-based programs that incorporate physical education and use culturally relevant practices to support adolescents in making healthy choices may enhance the feasibility and sustainability of the program. In order to develop locally relevant qualitative data to support the development of these types of strategies and to prevent the implementation of untested and inappropriate one-size-fits-all intervention models, there is a need to develop locally-relevant qualitative data.

### Strength and limitations

This study has several advantages, including theoretically-informed analytical framework, adherence to COREQ reporting guidelines, and representation of parents from various school settings. There are two primary limitations of this study. First, the sample size was consistent with qualitative research methodology, where the aim is to achieve in-depth understanding rather than statistical generalization. The sample size was guided by the principle of thematic saturation, ensuring adequate depth and richness of data. However, as the study was conducted within a specific geographic region (Coastal Karnataka), the findings may have limited transferability to other settings with different socio-cultural or educational contexts. Second, this study focused exclusively on parental perspectives and did not include the views of adolescents or teachers, who are key stakeholders influencing adolescents’ lifestyle behaviours. Adolescents’ perspectives may provide additional insights into their motivations, preferences, and behavioural choices, while teachers play a critical role in shaping school environments and opportunities for physical activity and dietary practices. The absence of these perspectives may limit the comprehensiveness of the findings. Future studies should adopt a multi-stakeholder approach to provide a more holistic understanding of the factors influencing adolescents’ lifestyle behaviours in similar contexts. Additionally, social desirability bias may have influenced parents’ reporting of their own and their adolescents’ healthy behaviors.

## Conclusion

Our qualitative study found that despite having a strong awareness of what constitutes a healthy lifestyle, maintaining a balance between PA and healthy dietary behaviours during adolescence is becoming increasingly challenging due to academic pressures, negotiated household practices, and socio-cultural influences. Especially in coastal Karnataka, where parents are experiencing rapid transitions in their educational and lifestyle environments, parents are facing a multitude of challenges to balance health promotion with the academic and social expectations of their adolescents. The findings of this study suggest a need for qualitative research in multiple contexts to guide adolescent chronic disease prevention programs that are culturally appropriate, family centered, and responsive to local environmental conditions. Ultimately, reducing the risk of chronic diseases in adolescents will depend upon the development of multi-level interventions that address the structural and contextual barriers that influence the daily behaviors of adolescents.

## Supplementary Information


Supplementary Material 1.



Supplementary Material 2.


## Data Availability

The datasets used and/or analysed during the current study are available from the corresponding author on reasonable request.

## Abbreviations

COM-B: Capability-Opportunity-Motivation Behaviour
CTRI: Clinical Trials Registry of India
COREQ: Consolidated Criteria for Reporting Qualitative Research
LMIC: Low- and middle-income countries
NCDs: Non-communicable diseases
PA: Physical Activity
SCT: Social Cognitive Theory
TPB: Theory of Planned Behaviour
