# Supplementary material for: Parental perspectives on physical inactivity and unhealthy diet as linked Non-Communicable Disease (NCD) risk factors among adolescents in Coastal Karnataka, India: a qualitative study
Source: BMC Public Health. 2026 Jun 22;26:2506. doi: 10.1186/s12889-026-28158-7 (PMC13536728; doi:10.1186/s12889-026-28158-7)
Supplement: Supplementary file 2 [file 12889_2026_28158_MOESM2_ESM.docx]

**Thank you for joining us today. We’re discussing how parents perceive the importance of adolescents' nutrition in relation to the risks of non-communicable diseases (NCDs). There are no right or wrong answers—we value your honest opinions.**

1. Can you share your family’s daily food habits, especially what your adolescent typically eats during the day?

Probes:

- Meal pattern- What does a usual breakfast, lunch, and dinner look like?
- What are the dishes that are regularly made at your house?
- Snacking habits- Are snacks or outside food part of their routine? Packed food like chips, cakes, chakuli and soft drinks?
- How often do you order take-out food as a meal for your family?
- What does your child's regular tiffin box comprise of?
- Do you cook tiffin for each day? Could you share a week’s plan with us if you could?
- How often does your child eat from the school/college canteen?
- In case you provide them with money for lunch at school, how much money do you give? What food items do you encourage/ recommend your child to eat in the canteen?
- How often do children eat fish, eggs, or meat? Do some families avoid them?
- Is fish (especially small fish like Boothai, Peethala) eaten whole for calcium and iron? Which kind of fish is often cooked in your house?
- How is it usually prepared? -Curry, fry?
- Which kind of fish is liked by your child?
- How often does your child eat chicken in a week? In what form? - biryani/ kababs/ lollipops/ curry/ sukka?
- Do you prepare it at home? How often do you order takeout, or does your child eat outside? - Which kind does your child like the most?
- Are eggs part of meals? If not, could you share the reason?
- Being a vegetarian, how and why do you encourage your child to eat eggs? How many in a day and is it included in which meal?
- How do you manage the protein intake of your child in each meal regularly?
- For vegetarians, does your child eat enough dal, sprouts, Paneer, and mushroom- which kind?
- What do you think is a good source of protein?
- How about green leafy vegetables?
- Does your child usually watch TV or use a mobile device while eating, and how does this affect their eating habits or attention during meals?
  2. What is your understanding of good nutrition and its importance for adolescents? What kind of food items do you think are mandatory and cannot be compromised?

Probes:

- Healthy/ unhealthy foods - What foods do you consider healthy or unhealthy?
- Balanced diet definition - How do you define a balanced diet for your adolescent?
- Do you provide extra supplements for nutrition? calcium Sandoz, cod liver oil, Bournvita/ Horlicks
- How often does your child fall sick because of stomach upset?
- What is the ideal meal preparation?
- Cold and cough? Kashaya?
- Worms?
- Regular water intake?
  3. How do you think nutrition affects your childs growth and long-term health?

Probes:

- Healthy diet- Can you describe what a healthy diet looks like for your child?
- Fullness vs nutrition- How do you balance fullness with nutritional value?
- Future health concerns- Are you concerned about future health issues like diabetes or obesity?
- What worries you most when your child skips breakfast before school?
- How do you manage your child’s food consumption during sports day or annual day?
- Have you noticed your child experiencing late-night or midnight food cravings, and how do you usually respond to them?
  4. How do you manage your adolescent’s nutritional needs alongside your daily family routines?

Probes:

- Time constraints- What role do time or work constraints play in your food choices?
- Affordability- Do costs or affordability influence what you can provide?
- Child preferences- How do your child’s food preferences factor into what you cook?
- Feeding challenges- Have you faced challenges like allergies or picky eating?
  5. Where do you go for advice or information about your child’s nutrition?

Probes:

- Information sources- Have you consulted healthcare providers, schools, or online resources?
- Nutrition knowledge- Do you feel well-informed about healthy eating for adolescents?
  6. Can you describe what mealtimes are like in your home?

Probes:

- Meal preparer- Who usually prepares meals? Who usually shops for food items/ groceries for the house?
- How often do you shop for groceries? Which oil, quantity and how often do you shop for it no of person in the family? Is coconut (oil, chutney, in curries) still widely used, or are refined oils replacing it?
- What are the typical food items you look out for, particularly for your child’s health?
- Cultural traditions- Do you follow any cultural or family traditions around food? (preparing some kind of foods during the time of festivals, having any one of the meals together with family members, no onion, garlic on any particular days as such)
- Are traditional sweets (like undae, sajjige) still made at home, or are biscuits replacing them?
  7. How do you make food-related decisions for your adolescent?

Probes:

- Meal planning- Do you plan meals or decide daily?
- Adolescent involvement- How much input does your adolescent have?
- Food disagreements- How do you manage disagreements about food?
  8. Are there specific foods or food groups you try to include or avoid in your adolescent’s diet?

Probes:

- Can you describe your child’s caffeine intake on a typical day, including sources such as tea, coffee, energy drinks, or soft drinks?
- Beverages and processed foods- What are your views on sugary drinks, processed snacks, or fast food? How often does your child demand and eat these food items in a week?
- Healthy food choices- Do you consciously try to increase fruits, vegetables, or whole grains? In what ways? Any preparation plans?
- Health concerns- Are any of your food decisions influenced by concern about NCDs?
  9. What makes it harder for you to provide healthy meals?

Probes:

- Difficult situation- Can you give an example of a difficult situation related to healthy food? What is the diet plan when you travel for holidays with your child? How often do you travel?
- Food marketing- Does marketing of unhealthy foods affect your child’s choices?
- Financial barriers- Are there logistical or financial barriers you face?
  10. How do media and technology affect your adolescent’s eating habits?

Probes:

- Social media - Have you noticed social media trends influencing what they eat?
- Advertisements - Do advertisements or screen time affect food timing or preferences?
  11. How does your community or school environment shape your child’s food habits?

Probes:

- Food options nearby- What food options are available near your home or school?
- School food distribution- How would you describe the food sold or served at school?
- Cost and availability- Do availability and cost impact your food choices?
  12. How do relationships with others influence your adolescent’s eating habits?

Probes:

- Peer/Teacher Influence- How do peers or teachers’ impact what your child eats? Peer group going out
- When your child goes out to eat with friends, what kinds of foods do they usually choose, and what influences their choices? And how often do they tend to eat outside?
- Parental Influence- Do you feel your habits influence theirs?
- Socioeconomic Impact- Does your socioeconomic status affect the food choices available to you?
- Does your child idealize any social media influencer or celebrity for food-related references?
- Does your child follow or is fascinated with any kind of special eating regimen like the Keto diet/ high protein diet/ intermittent fasting?
- Is your child body conscious? Boys- muscles, toned body. Girls- flat stomach, lean body etc
- What are their regular complaints related to food intake?
- Could you describe a few instances?
- Do they demand food to be made a particular way? Any specific food items?
  13. What personal factors do you think influence your adolescent’s food choices?

Probes:

- Appearance- Do they express concerns about appearance or body image?
- Stress impact- How does stress or school pressure affect their eating? During exam periods, have you noticed any changes in your child's caffeine intake, binge eating or avoid food? how do you think it affects their focus or sleep? How do you address them?
- Gender differences- Do you see differences between boys and girls in your family?
  14. How has your adolescent’s growing independence affected how and what they eat?

Probes:

- Independent food choices- Are they buying or choosing food on their own now?
- Parents' feelings- How do you feel about their choices?
- Supporting independence- How do you support healthy habits while giving them independence?
- Do you ask about your child's food consumption and frequency?
  15. Thinking about all these influences, how concerned are you about your adolescent’s risk for health problems like diabetes or obesity?

Probes:

- Change in diet- Are you trying to change any eating habits at home?
- Helpful resources- What kind of help or information would be most useful?
- Parental assurance- Do you feel confident about influencing your child’s eating habits?

**Introductory access:**

**Start of Interview:**

**"Thank you for joining us in this interview today. We are discussing how adolescents engage in physical activity and what is the role of parents in supporting this. There are no right or wrong answers – we respect your honest opinions. We're going to ask you a few questions to understand the basic purpose of this."**

**1. Please tell me your name. What is your child's name and age?**
**2. What activity do they enjoy in their free time?**

**Key Questions:**

**About Physical Activity:**

3. What is the first thing that comes to your mind when you hear of 'physical activity' for a teenager?
4. What does "enough" physical activity mean in your opinion?
5. Do you think your child is quite physically active? Why/why not?
6. What are the activities that you consider to be physical activity? (E.g. playing, homework, walking to school)
7. Why do you think physical activity is important for your child?
8. What cultural activities do you think are physically difficult? (e.g. Yakshagana, Yoga, Karate)

**About Unique Activities (Yakshagana/Yoga/Martial Arts)**

**If it is Yakshagana**

9. When you think of adolescent physical activity, do traditional practices like Yakshagana/Yoga or Karate come to your mind? Why/why not?
10. Does your child practice this? How many hours a day and how many times a week? And when did they start this?
11. What is your opinion about the physical demands of these activities/what do you think there will be physical challenges (e.g. dance, stamina, costumes)?
12. Do you think these activities are more suitable for boys or girls? Why?
13. Can you have gender-based ideas in these activities? (E.g. Yakshagana is only for boys)
14. Do you think activities like Yakshagana or yoga help teenagers connect with their culture?

**Yoga-related questions:**

14. Do you encourage yoga for your child? How do you see its benefits (for example, maintaining a healthy weight, flexibility, mental focus)?
15. Can you explain what asanas your child practises, how many hours a day and how many times a week? And when did they start this?
16. In what way is yoga seen in your home (as an 'exercise' or a spiritual/cultural practice)?
17. Is yoga part of the curriculum in your child's school? Do you think that's necessary? Could you please explain to me on this?

**Martial arts (karate, silambam):**

18. Are these activities popular in your community? What skills do you think teenagers will develop/develop from these practices?
19. Does your child practice this? How many hours a day and how many times a week? And when did they start this?
20. Do you believe that karate helps with discipline or self-defense? How?

**Comparison of Modern Sports and Traditions:**

21. Compared to modern sports, how do you feel about your child participating in these traditional activities?
22. Do you prefer Yakshagana/Yoga/Martial arts over cricket or football? Why/why not?
23. Do schools or community centres promote these traditions enough?
24. How do these activities compete with screen time or other modern sports?

**Social Media and Influences:**

25. Is your child motivated by any celebrity or social media influencer for physical activity?
26. Do you think celebrities and social media are helping to make yoga more popular and accepted? What is your understanding?

**Emotional Influences:**

27. How does physical activity affect your child's state of mind or stress level?
28. Have you noticed any changes in health or behaviour when they are more or less active?
29. Do you think there is a link between physical activity and the effects on their mental health or studies?

**Impact of Environment and Habitat:**

30. Does living in a coastal Karnataka (urban or rural) area have any impact on your child's physical activity?
31. Are there cultural or community expectations related to participation in the activity?
32. Are there safe places to play? Are some activities considered suitable for boys/girls only?
33. How do schools or local facilities support/hinder physical activity?

**Parent's View:**

34. How do you encourage or support your child to be physically active?
35. What challenges do you face in this? (E.g. lack of time, fear of safety, pressure to read)
36. Are there differences in supporting your son/daughters?
37. How will you support the child to get involved in these activities? (e.g. enrolling in classes, taking them to demonstrations at events)
38. Which of these challenges are hindering this participation? (Cost, time or social pressures)
39. Are these classes or teachers readily available in your area? (For example, trained teachers, affordable classes)

**Finally:**

40. What's one thing you can change to help your child be more active?
41. Is there anything else you'd like to share on this topic?

**Conclusion: Thank you for your valuable insights! Your feedback will help us understand how to better support the health of adolescents in our community.**
